# Heterotopic Pregnancy Following Ovulation Induction With Successful Pregnancy Outcome: A Case Report

**DOI:** 10.1002/ccr3.70277

**Published:** 2025-02-25

**Authors:** Urbi Ghimire, Isha Manandhar, Samiksha Shrestha, Rambha Sah, Jyoti Lamichhane, Jageshwor Gautam

**Affiliations:** ^1^ Om Hospital and Research Center Pvt. Ltd Kathmandu Nepal; ^2^ Department of Gynecology and Obstetrics Om Hospital and Research Center Pvt. Ltd Kathmandu Nepal

**Keywords:** clomiphene, ectopic, heterotopic, pregnancy

## Abstract

Heterotopic pregnancy (HTP) is a rare, life‐threatening condition in which both extrauterine and intrauterine gestation co‐occur. It presents diagnostic and therapeutic challenges for physicians, often being missed or overlooked. A heterotopic pregnancy must always be considered if a patient presents with pelvic pain, especially when pregnancy is aided with ovulation‐inducing agents or assisted reproductive technologies (ARTs).

## 1 Introduction

Heterotopic pregnancy (HTP) is the coexistence of two or more pregnancies at different implantation sites [1]. It is an uncommon complication of pregnancy in which both extrauterine and intrauterine gestation can coincide [2]. According to the literature, the incidence of HTP in spontaneous pregnancy is estimated to be 1/30,000 [1]. However, a rate of 1/100 is reported with assisted reproductive technologies (ARTs) [3, 4, 5] and 1/900 in pregnancies aided with Clomiphene Citrate [4]. This shows that the cases of HTP are higher with ART and comparatively lower in spontaneous and clomiphene‐induced pregnancy [5]. HTP might induce a life‐threatening complication of pregnancy, and its diagnosis is often difficult. Transvaginal ultrasound is the gold standard for diagnosis despite its low sensitivity, and plasmatic beta‐human chorionic gonadotropin (beta‐hCG) is often not considered as hormonal imbalances are masked by intra‐uterine pregnancy (IUP) [6, 7, 8].

This study describes a case of HTP conceived with clomiphene citrate. The pregnancy was continued till term following Right laparoscopic Salpingectomy and a healthy baby was delivered.

## 2 Presentation of Case

### 2.1 Case History/Examination

A 28‐year‐old woman (G3P1 + 1 L0) came to the OPD following cessation of menses for 2 months for which she used a home pregnancy test kit and the results were positive. The patient was under Tab. Clomiphene citrate (50 mg) 2 tables [D2–D6] for ovulation induction. She gives a history of PV spotting (minimal) for 10 days, continuous, associated with lower abdominal pain, however, her vitals were stable during the time of the visit. She has a previous history of Left Laparoscopic Salpingectomy for ruptured left tubal ectopic pregnancy and also a neonatal death following pneumonia after a premature delivery at 7 months.

**FIGURE 1:**
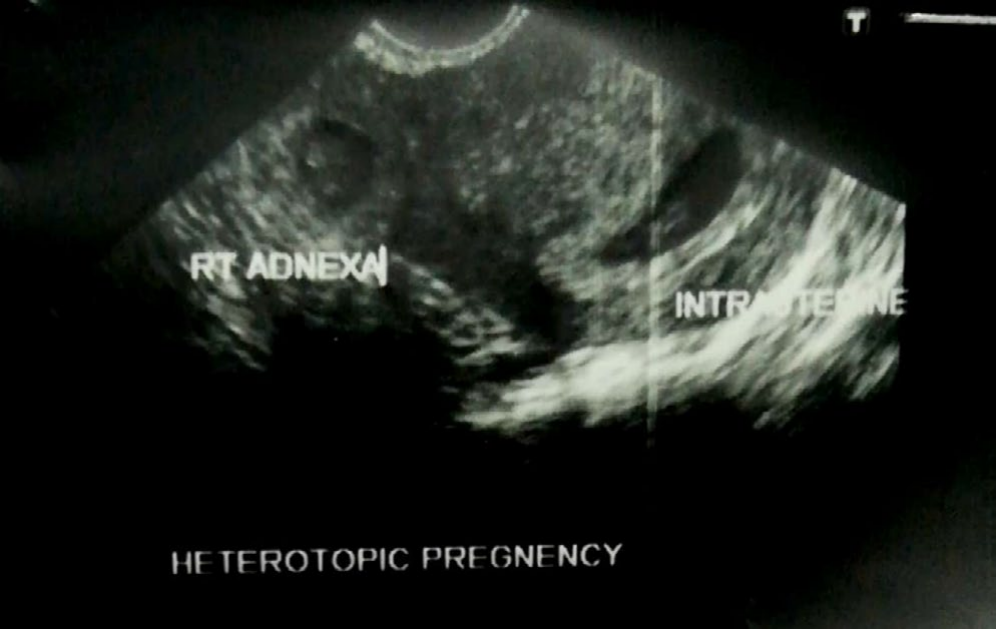
A case of Heterotopic pregnancy with intrauterine and extrauterine gestation.

A USG‐TVS was advised, which showed:

1. Heterotopic pregnancy in the uterine cavity and right para‐uterine region (Figure 1)
2. Intrauterine pregnancy of 6 weeks 3 days with cardiac activity
3. Ectopic pregnancy in right para‐uterine region of 6 weeks 5 days with no cardiac activity

## 3 Methods

**FIGURE 2:**
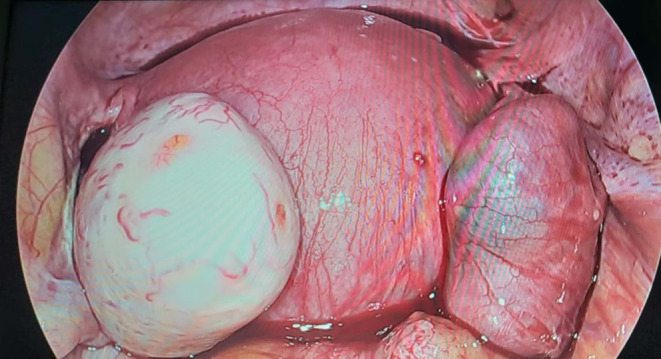
Tubal ectopic pregnancy with 6 weeks of intrauterine gestation.

**FIGURE 3:**
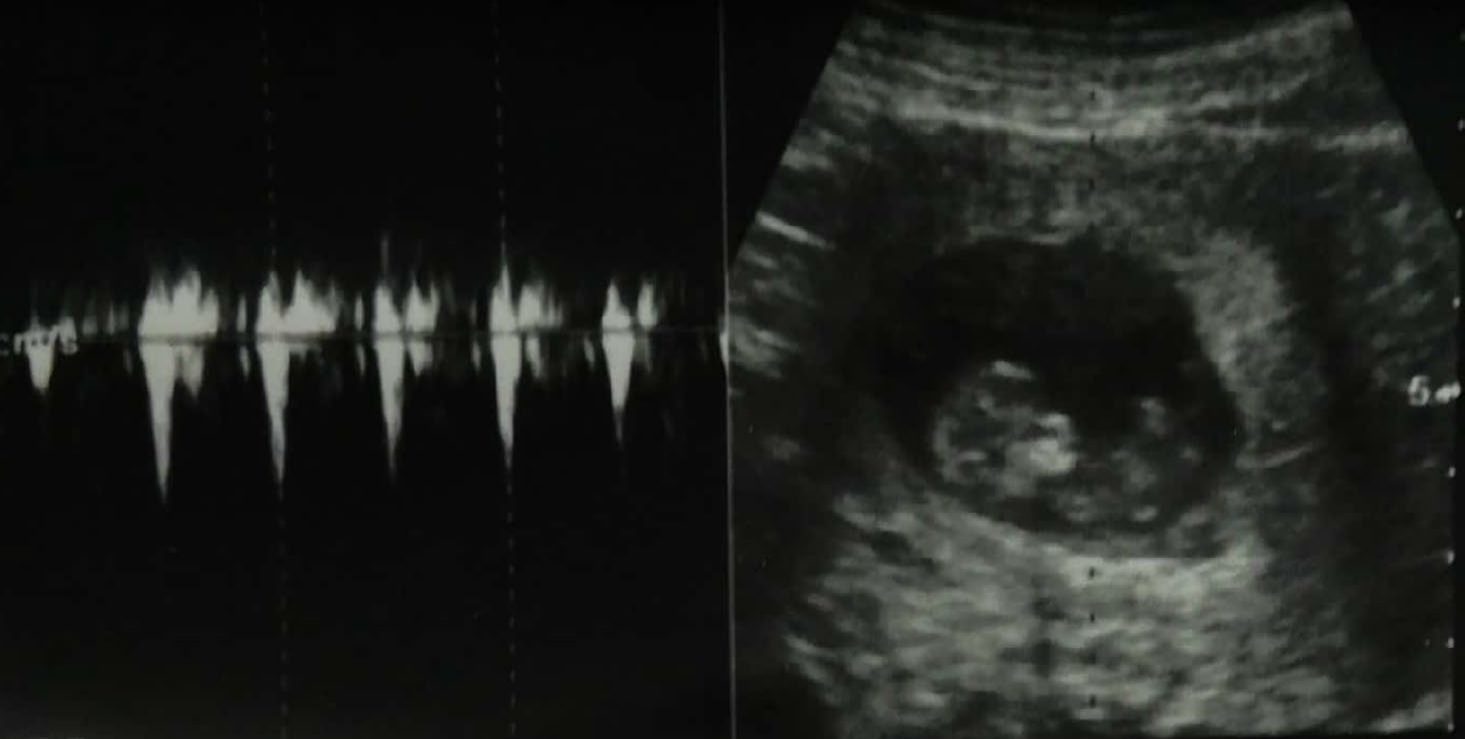
Continuation of IUP following salpingectomy.

After careful counseling, the patient was prepared for emergency surgery for right tubal ectopic pregnancy. A written informed consent was taken and a right laparoscopic salpingectomy was done (Figure 2). During surgery, a hemoperitoneum of ~50 mL was found and was cleaved. Both the ovaries were found to be normal, however, the left tube was not visualized due to a previous left salpingectomy for a tubal ectopic pregnancy. The intrauterine pregnancy was unharmed and continued till term (Figure 3) Her blood reports were normal before and after the surgery, and the antenatal blood investigations were non‐significant. The postoperative period was non‐remarkable, and the patient was stable. Thus, she was discharged on 1st postoperative day. The patient came for a follow‐up after a week and the postoperative USG showed an intrauterine gestation of 7 weeks, which was later continued till term.

She came for a regular antenatal checkup. She was under Tab. Folic acid and Cap. Gestofit (micronized progesterone) in 1st trimester. Tab. Iron and Tab. Calcium were given till 6 weeks postpartum. Two doses of tetanus were taken in 4th and 5th month. Anomaly scan was done at 20 weeks, which showed no abnormalities. She was diagnosed with gestational diabetes mellitus (GDM) and hypothyroidism at 27 weeks of gestation (WOG) and was consulted with a physician and thus, was started on Tab—metformin‐500 mg OD and Tab Thyroxin 25mcg OD. After 2 weeks, following the reports of thyroid function tests (TFT) and fasting blood sugar (FBS), the medicines were upgraded to Tab. Metformin‐ 1 g OD and Thyroxin 25mcg OD 5 days a week and 50mcg OD 2 days a week.

The pregnancy was continued till term (37 + 6 WOG) following which an emergency lower section cesarean section (LSCS) was performed for premature rupture of membrane (PROM) with Severe Oligohydramnios (AFI‐2). A healthy female baby weighing 2.76 kg was delivered on 2023/10/25.

## 4 Outcome and Follow‐Up

The patient and the baby were discharged on the third postoperative day. The vitals of the mother and baby were stable, and the baby was breastfeeding well. She was prescribed antibiotics, iron, and calcium during discharge and was called for a follow‐up in a week.

During follow‐up, the wound of the mother was healthy. The baby was playful and was breastfeeding well. Iron and Calcium were continued till 45 days postpartum.

## 5 Discussion

The coexistence of two or more pregnancies at separate implantation sites is known as Heterotopic pregnancy [1]. The incidence of Heterotopic pregnancy in spontaneous pregnancy is estimated to be 1/30,000 [1]. However, the incidence has surged with the rise in the use of assisted reproductive technologies, reaching rates of 1/100 in the population [3, 4, 5]. Clomiphene Citrate is associated with a heterotopic pregnancy rate of 1/900, which is significantly lower than that achieved with assisted reproductive technology [4, 5, 8]. HTP mostly occurs with certain risk factors such as assisted reproduction, ovulation induction, and a history of ectopic pregnancy or pelvic inflammatory illness [1, 8]. In our case, the implantation sites were the uterus and the right‐sided para‐uterine region. Also, a history of left‐sided tubal ectopic pregnancy was present a few months prior, for which a laparoscopic salpingectomy was done.

Duverney reported the first naturally occurring heterotopic pregnancy in 1708 during an autopsy [9, 10]. Since then, hundreds of reports on heterotopic pregnancies have been published, 589 of which were reviewed by Reece et al. In 1971, Payne et al. documented the first case of heterotopic pregnancy following clomiphene citrate (CC) and corticosteroid treatment, where the patient presented with uterine pregnancy with the signs and symptoms of a ruptured EP [9].

The preoperative diagnosis of HTP remains a major challenge as most patients are asymptomatic and are detected extremely late, with severe morbidity and mortality [7, 8]. In our case, the patient presented with lower abdominal pain and vaginal bleeding, and the ectopic gestation was unruptured. Plasmatic beta‐human chorionic gonadotropin (beta‐hCG) level was not done in our patient. It is often difficult to interpret because the subnormal hormone production by an ectopic pregnancy may be masked by the higher placental production from the intrauterine pregnancy [6, 7, 8].

In a low‐risk, asymptomatic patient with ovulation induction, diagnosis of HTP via USG can be extremely challenging [7, 8, 11]. However, USG‐TVS helped to diagnose our case. In a literature review of all cases of HTP from 1971 to 1993, 46 cases out of 112 were diagnosed by USG, while the rest of the 66 cases were diagnosed during laparoscopy or laparotomy. The recent literature analysis from 1994 to 2004 also revealed that USG helped to diagnose 21 out of 80 cases, and the rest of the 59 were detected during laparoscopy or laparotomy [7]. Despite its low sensitivity (33%) in detecting HP, transvaginal ultrasound is the gold standard for diagnosis [1].

Heterotopic pregnancy is more likely to end in spontaneous (relative risk = 2.05; 95% confidence interval, 1.67–2.51) or induced (relative risk = 10.28, 95% confidence interval, 6.76–15.65) abortion than intrauterine‐only pregnancy [12]. In our case, after the tubal ectopic gestation was removed during 6 weeks of gestation, the IUP was continued until term.

The therapeutic options are mostly surgical, either by laparoscopy or by laparotomy [3, 13]. The review by Talbot et al. states that 33% of HTP patients are hemodynamically unstable, making laparotomy mandatory [3, 14]. However, a laparoscopic procedure is more appropriate if the patient is hemodynamically stable [3]. As our patient was hemodynamically stable, we opted for laparoscopic surgery as well. The intrauterine pregnancy was attempted to be preserved while the tubal ectopic pregnancy was removed during surgery. Low pneumoperitoneum pressure is necessary for laparoscopy, following needle insertion far from the gravid uterus [3].

A heterotopic pregnancy is a diagnostic and therapeutic challenge. Although extremely rare, it should always be kept in mind, even if an intrauterine pregnancy is diagnosed, especially if the pregnancy is conceived with the aid of ovulation‐inducing agents or assisted reproductive technologies. Evaluation of adnexa using abdominal and transvaginal ultrasonography should always be performed, as it can be missed otherwise.

## Author Contributions

**Urbi Ghimire:** conceptualization, data curation, formal analysis, investigation, resources, writing – original draft. **Isha Manandhar:** writing – review and editing. **Samiksha Shrestha:** writing – review and editing. **Rambha Sah:** writing – review and editing. **Jyoti Lamichhane:** supervision, writing – review and editing. **Jageshwor Gautam:** supervision, validation, visualization.

## Ethics Statement

Our institution does not require approval from the Ethics Review Committee for case reports.

## Consent

Written informed consent was obtained from the patient to publish this case report and accompanying images, per the journal's patient consent policy.

## Conflicts of Interest

The authors declare no conflicts of interest.

## Data Availability

The data that support the findings of this study are available from the corresponding author upon reasonable request.
